# Epidemiological and Clinical Characteristics of Patients Admitted to a Secondary Hospital with Suspected MPOX Virus Infection: Is HIV Playing a Role?

**DOI:** 10.3390/jcm12124124

**Published:** 2023-06-18

**Authors:** Samuel Estévez, Marta Vara, María Gamo, Samuel Manzano, Jesús Troya, Ekaterina Botezat, Eva Jiménez, Roberto Pedrero-Tomé, María Ángeles Martin, Pablo de la Cueva, Elisa Fernández, Beatriz Fernández, David Esteban Brown, Elena Palma, Ana Simón

**Affiliations:** 1 Department of Internal Medicine, Infanta Leonor University Hospital, 28031 Madrid, Spain; 2 Department of Preventive Medicine and Public Health, Infanta Leonor University Hospital, 28031 Madrid, Spain; 3 Department of Dermatology, Infanta Leonor University Hospital, 28031 Madrid, Spain; 4 Foundation for Biomedical Research and Innovation (FIIB), Infanta Leonor University Hospital, 28031 Madrid, Spain

**Keywords:** MPOX, smallpox, HIV, PLWHIV, MSM

## Abstract

MPOX (monkeypox) is a zoonotic viral disease, endemic in some Central and West African countries. However, in May 2022, cases began to be reported in non-endemic countries, demonstrating community transmission. Since the beginning of the outbreak, different epidemiological and clinical behaviors have been observed. We conducted an observational study at a secondary hospital in Madrid to characterize suspected and confirmed cases of MPOX epidemiologically and clinically. Besides the general descriptive analysis, we compared data between HIV-positive and HIV-negative subjects; 133 patients were evaluated with suspected MPOX, of which 100 were confirmed. Regarding positive cases, 71.0% were HIV positive, and 99.0% were men with a mean age of 33. In the previous year, 97.6% reported having sex with men, 53.6% used apps for sexual encounters, 22.9% practiced chemsex, and 16.7% went to saunas. Inguinal adenopathies were significantly higher in MPOX cases (54.0% vs. 12.1%, *p* < 0.001), as the involvement of genital and perianal area (57.0% vs. 27.3% and 17.0% vs. 1.0%, *p* = 0.006 and *p* = 0.082 respectively). Pustules were the most common skin lesion (45.0%). In HIV-positive cases, only 6.9% had a detectable viral load, and the mean CD4 count was 607.0/mm^3^. No significant differences were observed in the disease course, except for a greater tendency towards the appearance of perianal lesions. In conclusion, the MPOX 2022 outbreak in our area has been related to sexual intercourse among MSM, with no severe clinical cases nor apparent differences in HIV and non-HIV patients.

## 1. Introduction

MPOX is a viral disease caused by a double-stranded DNA virus of the genus Orthopoxvirus, family Poxviridae [1]. It was discovered in 1958 in Cynomolgus monkeys [2]. MPOX primarily infects small mammals and apes, with small rodents suspected as the primary natural reservoir [3].

Its zoonotic capacity was confirmed in 1970 when transmitted to a 9-month-old child in the Democratic Republic of Congo (DRC) [2]. Human-to-human transmission mainly occurs through close contact with skin lesions and crusts [4]. Droplet transmission through prolonged face-to-face contact is also possible; however, the risk of airborne transmission is minimal [5]. The virus can also be transmitted via fomites and the transplacental route [4]. In addition, human-to-animal transmission has recently been confirmed [6,7]. 

The disease is usually self-limiting, although clinical sequelae are common [8]. After a prodromal phase characterized by fever, myalgia, lymphadenopathy, and headache, a skin eruption follows 2–4 days later, with lesions at different stages (macules, papules, pustules, and scabs) distributed on the face and body, including oral and genital mucosa [9]. The main complications of this entity include bacterial superinfection, bronchopneumonia, encephalitis, and keratitis [10]. Higher-risk populations include neonates, children, and those with immunodeficiency [11]. Although there are limited data among people living with HIV (PLWHIV), it appears that those with persistent detectable viral load, low CD4 count, or a recent HIV-related illness are more likely to develop complications [12]. The treatment of this zoonosis is essentially symptomatic [13]. Among other preventive measures, pre- and post-exposure vaccination strategies in certain risk groups have been implemented, essentially with the 3rd-generation vaccine of modified live Ankara vaccinia virus [14].

MPOX is endemic in some Central and West African countries, where transmission is mainly zoonotic or through close contact in family settings. However, there has been an increase in cases in the last three decades [8]. Moreover, four major human outbreaks, including this one, started in 2022 [3].

On 7 May, the UK reported a case of MPOX, imported from Nigeria, quickly diagnosed at arrival; no connection was found with subsequent cases, despite tracing contacts [15]. The UK announced new cases on 13 and 15 May [16], spreading in the following week to several countries in Europe, Canada, and the USA [17]. On 23 July, the WHO declared the outbreak a Public Health Emergency of International Concern (PHEIC). The outbreak is spreading mainly among men, especially men who have sex with men (MSM) [18]. There are no signs suggesting sustained transmission beyond this setting. Ten months later, the number of cases has declined substantially, but WHO considers the global risk moderate, and hence the PHEIC has been maintained [19].

There are two distinguished clades of the virus. Clade I, from Central Africa, has a mortality rate higher than 10% and has higher transmissibility. Clade II has a mortality rate of less than 1% and has been further subdivided into clades IIa (prevalent in West Africa) and IIb (sequenced in the Nigerian outbreak of 2017, still ongoing, and the current outbreak of 2022) [3].

Given the need to increase knowledge about this recent outbreak of MPOX in non-endemic countries, this article aims to summarize the main epidemiological and clinical findings of our observational study conducted in a hospital in Madrid, Spain. In addition, we will highlight the possible differences between confirmed cases and those that were finally ruled out, and between PLWHIV and HIV negative individuals.

## 2. Materials and Methods

We conducted an observational analysis of all the patients with suspected, probable, or confirmed MPOX virus infection admitted to Hospital Universitario Infanta Leonor from 18 May to 30 September 2022.

The Spanish Ministry of Health carried out a “Protocol for the early detection and management of monkeypox cases in Spain, in which definitions of suspect, probable, and confirmed MPOX cases were proposed. A suspected MPOX case is defined by an individual clinically presenting with a localized or generalized rash (with vesicular or pustular lesions, especially if they are umbilicated) and one or more of the following signs/symptoms: fever (>38.5 °C), severe headache, myalgia, arthralgia, backache or enlarged lymph nodes. A probable case is defined when a subject, apart from meeting the previous clinical criteria, fulfills epidemiological criteria, namely one or more of the following: contact with a probable or confirmed MPOX case within 21 days before the onset of symptoms, sexual intercourse with multiple or anonymous random sexual partners within 21 days before symptom onset or travel history to MPOX endemic countries within 21 days of symptom onset. Finally, a confirmed case is defined as a subject with a laboratory-proven MPOX infection (in a clinical sample, using a reliable and validated technique such as a polymerase chain reaction [PCR] test) using similar criteria to those used in other European countries. In our study, MPOX was confirmed using a real-time PCR test (RT-PCR) by Roche (specific target of Monkeypox), Altona (target Orthopoxvirus and Variola virus), Progenie (specific target of Monkeypox) or a generic one in accordance with Hugget, Eurosurvilliance 2022 [20]. All techniques were evaluated and compared prior to implementation.

### 2.1. Objectives

The main objective of the study was to clinically and epidemiologically characterize the MPOX suspected and confirmed cases. Secondly, we compared HIV-positive and HIV-negative case data to describe possible clinical and epidemiological differences.

### 2.2. Methodology

Patients were evaluated by an interdisciplinary team of infectious disease specialists, dermatologists, emergency medicine specialists, public health, and preventive medicine specialists. Initially, patients admitted to the hospital were assessed by an emergency medicine specialist, an infectious disease specialist, or a dermatologist who performed a standard medical examination and diagnosed suspicion. Then, a viral swab was taken from a skin lesion to demonstrate MPOX DNA by a PCR test. Simultaneous screening for sexually transmitted infections (STI) was carried out. Samples for the confirming infection were sent and processed at the National Centre of Microbiology. The preventive medicine specialist obtained sociodemographic data. Confirmed cases were jointly evaluated by the dermatologist and infectious disease specialist for shared management and follow-up.

### 2.3. Data Collection

Data collected included sociodemographic and epidemiological features, such as gender and sexual orientation, HIV serology, sexual health data, epidemiological exposures, clinical symptoms and signs observed during the examination, mucocutaneous manifestations, admission to hospital, and final diagnosis. In addition, the evolution of lesions and possible complications were collected from some of the confirmed cases. Clinical photographs of lesions were also documented after obtaining patients’ written informed consent.

Study data were collected and managed using Microsoft Excel version 16.62 and the data capture tool Research Electronic Data Capture (REDCap), which is hosted at “Association Ideas for Health” [21]. The study protocol was formally approved by the Ethics Committee of Hospital Universitario Infanta Leonor and fulfilled the principles of the Declaration of Helsinki (ethical approval code 22/423-E).

### 2.4. Statistical Analysis

A descriptive study of the collected variables was carried out. The sample was described using absolute and relative frequencies for categorical variables and median and interquartile range (IQR) for continuous variables. No imputation was made for missing data.

In addition to descriptive statistics, tests were carried out to determine differences between confirmed MPOX cases and patients with negative test results and between HIV-infected MPOX cases and HIV-negative MPOX cases. χ^2^-tests for qualitative variables and *t*-tests for quantitative variables were used. For data not fitting the normal distribution, non-parametric tests were applied. All tests were two-sided with a significance threshold of 0.05. The statistical procedure was performed using the R Core Team software (version 4.3.1, 2022).

## 3. Results

### 3.1. Epidemiological Features

One hundred and thirty-three patients were evaluated at our hospital with suspected MPOX, and the diagnosis was confirmed in one hundred of them. Regarding positive cases, 99% were male, with a median age of 33 years (IQR 30.0–42.0). Most had at least a secondary school education, and all reported low to medium socioeconomic levels. Regarding sexual health and practices, 54.5% did not have a steady sexual partner, and 14.8% reported being in an open relationship. In the previous 12 months, 97.6% of the confirmed MPOX cases reported having sex with men (MSM), 16.7% went to saunas, 53.6% used an app for sexual encounters, 22.9% practiced chemsex, and 6.0% had sex with sex workers. The number of different sexual partners in the last 12 months, frequency of condom use, and other epidemiological information are summarized in Table 1.

### 3.2. Clinical Features

#### 3.2.1. MPOX-Confirmed Cases vs. Negatives

**Figure 1:**
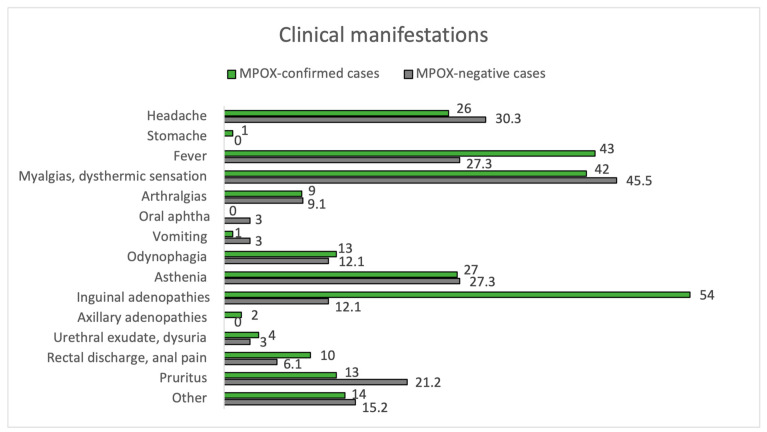
Clinical manifestations. MPOX-confirmed vs. MPOX-negative cases.

**Figure 2:**
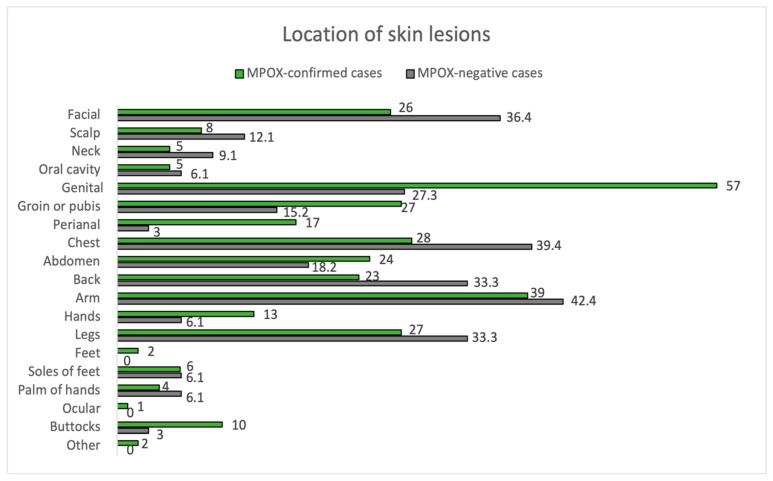
Location of skin lesions. MPOX-confirmed vs. MPOX-negative cases.

Of all positive cases, 98.9% met the criteria for a suspected case, while only 81.2% of negative cases did (*p* < 0.01). The clinical characteristics of the confirmed and negative cases are summarized in Figure 1 and Figure 2, and Table 2.

The mean time between symptom onset and the appearance of skin lesions was 1 day (±3 days). Inguinal adenopathy was significantly higher in the positive group (54.0% vs. 12.1%, *p* < 0.001). MPOX-confirmed cases had an increased prevalence of fever compared to negatives (43.0% vs. 27.3%), although they did not reach statistical significance (*p* = 0.162). Fever (43.0%) and myalgias (42.0%) were the most common prodromal symptoms in MPOX cases. Most participants in both groups had between 2 to 25 lesions (54.0% in MPOX-confirmed cases and 57.6% in negatives), but 12.1% of the negatives had >25 lesions, compared to only 1.0% of the confirmed cases (*p* = 0.017). In addition, 4.0% of MPOX cases had a solitary lesion. Overall, lesion size varied between 1–5 mm. Pustules were the most common skin lesion (45.0%) in MPOX-confirmed cases. Regarding the location of skin lesions, 57.0% of MPOX cases had genital involvement, and 17.0% had perianal lesions. In comparison, genital areas were affected only in 27.3% and perianal only in 1% of non-infected subjects (*p* = 0.006 and *p* = 0.082, respectively). Trunks and limbs were frequently affected in both groups, as can be seen in Figure 2.

The favorable evolution of skin lesions is most frequent, as seen in Table 2. Bacterial superinfection accounted for 9.0% of MPOX patients, with a remaining residual atrophic lesion in 6.0%, while no superinfection occurred in negative cases. Six percent of MPOX cases had to stay hospitalized for over one day. There was no mortality in either of the two groups. Alternative diagnoses in MPOX-negative patients were smallpox, syphilis, herpes virus, multiple insect bites, hand-foot-mouth disease, pityriasis rosea, scabies, and gonococcal proctitis. No pneumonia episodes have been diagnosed in either the PLWHIV or in HIV-negative patients.

#### 3.2.2. HIV-Infected Cases Description

Of the total number of patients, 62.1% were PLWHIV, corresponding to 82 cases. MPOX was confirmed in 71 cases (84.2%) and ruled out in 11 (13.4%). At HIV diagnosis, the median CD4 count was 284.0 cells/mm^3^ (IQR 192.0–462.0), 59.3% met late diagnostic criteria (CD4 count below 350 cells/mm^3^), and 9.1% had AIDS. At the time of MPOX diagnosis, the median CD4 count was 607.0 cells/mm^3^ [IQR 394.0–914.0], with only four patients (6.9%) having a detectable viral load. All HIV-patients were on antiretroviral treatment which included 51% with non-nucleoside reverse transcriptase inhibitors (efavirenz, nevirapine, rilpivirine), 36.7% with integrase inhibitors (dolutegravir, raltegravir, bictegravir, elvitegravir), and 12.2% with boosted protease inhibitors (darunavir, lopinavir, atazanavir). Only two patients were in dual therapy with dolutegravir/lamivudine. All other HIV-related data are summarized in Table 3.

The analytical results are summarized in Table A1, in Appendix A. C-reactive protein levels in confirmed MPOX cases with HIV were higher than those without HIV, with a median of 46.3 mg/L vs. 14.1 mg/L (*p* = 0.026).

#### 3.2.3. HIV-Infected MPOX Cases vs. HIV-Uninfected MPOX Cases

**Figure 3:**
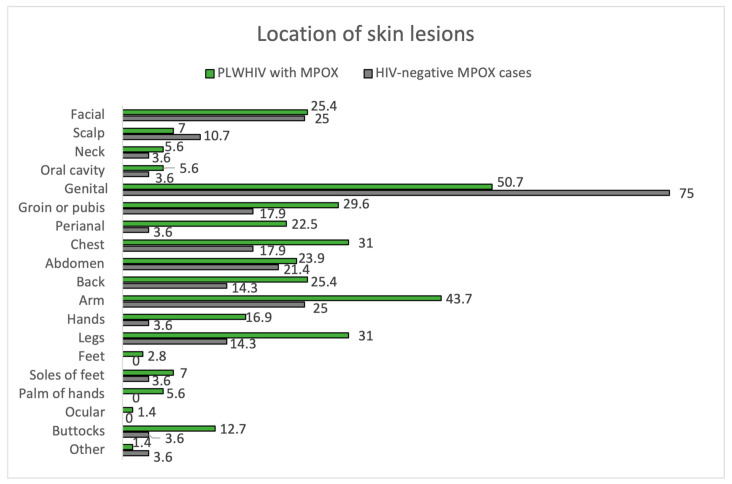
Location of skin lesions. PLWHIV with MPOX vs. HIV-negative MPOX cases.

Of the 100 MPOX cases, 71.0% were PLWHIV, and 28.0% were HIV-negative, considering one loss. In terms of skin manifestations, the number of lesions and their size were similar in both groups, with the most frequent being between 2 and 25 lesions and 1 to 5 mm in size. In both groups, the most frequently affected area was the genital area, as seen in Figure 3. There is a trend towards greater involvement of the perianal area among PLWHIV compared to non-HIV patients (22.5% vs. 3.6%, *p* = 0.05). This aspect is more remarkable in PLWHIV with poor control criteria (CD4 count < 500 cells/mm^3^), with perianal involvement in this group at 30.3% vs. 0.0% if they had accurate HIV control. Facial involvement among PLWHIV occurs more frequently among those meeting criteria for poor control (24.2% vs. 0.0%, *p* = 0.349). The course of MPOX was favorable in most patients in both groups. Of the nine patients with bacterial superinfection of skin lesions, eight were PLWHIV. Hospitalization was required in six patients, five corresponding to PLWHIV. None of the patients died from MPOX.

**Figure 4:**
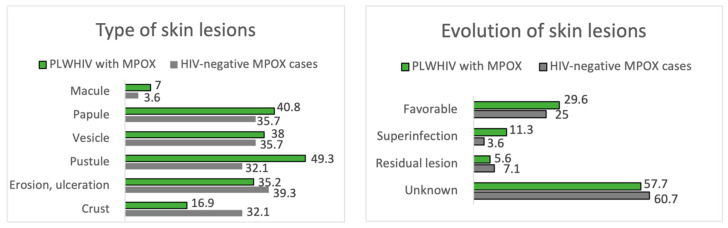
Type and evolution of skin lesions. PLWHIV with MPOX vs. HIV-negative MPOX cases.

**Figure 5:**
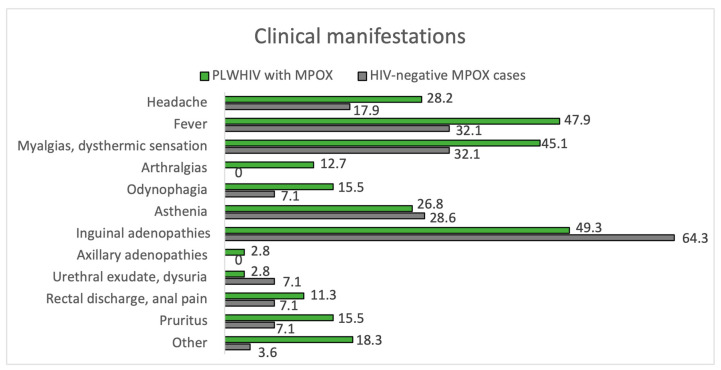
Clinical manifestations. PLWHIV with MPOX vs. HIV-negative MPOX cases.

The remaining results comparing the manifestations of MPOX between PLWHIV and HIV-negative patients are summarized in Figure 4 and Figure 5, Table 2 and Table 4.

#### 3.2.4. Smallpox-Vaccinated vs. Unvaccinated against Smallpox

Of the MPOX-confirmed cases, 15.0% were vaccinated against smallpox. Fever is less frequent among vaccinated patients, occurring in 14.3% vs. 51.6% among unvaccinated patients (*p* = 0.025). In both subgroups, most patients had between 2 and 25 lesions, 1 to 5 mm in size, with no notable differences. Skin lesions in vaccinated versus unvaccinated patients showed a tendency to appear less frequently on the abdomen (7.1% vs. 34.4%) and on the legs (7.1% vs. 35.9%) without reaching statistical significance in either case (*p* = 0.089 and *p* = 0.073 respectively). The most frequently affected location was the genital region, about 50% in both groups. The evolution of the skin lesions was favorable in most patients in both groups, but bacterial infection occurred in 8 of the 66 unvaccinated patients (12.5%) compared to the absence of this complication among the 15 vaccinated patients. Of the six patients who required hospital admission, none were vaccinated against smallpox, and the vaccination status of 1 of these patients is unknown. The remaining results comparing the manifestations of MPOX between those vaccinated and those unvaccinated against smallpox are summarized too in Table 4.

## 4. Discussion

### 4.1. Epidemiological Discussion

In the current outbreak in 2022, the occurrence of MPOX in several regions simultaneously, in the absence of epidemiological links to endemic areas, suggests possible silent transmission for some time [19]. As of this writing, it is remarkable that investigations into the frequency and circumstances under which the virus may be spread through respiratory secretions are still ongoing [6]. However, body fluids having the potential for transmission, including semen, vaginal fluids, urine, and feces, have not been definitively shown to be infectious [22].

Regarding demographic parameters of age and sex, it is noteworthy that the data found in our hospital exhibit significant similarities to those observed in other publications of the recent MPOX outbreak: the majority of cases were young adults (aged 30–42 years) and occurred in MSM. For example, the Spanish Health Emergency and Alert Control Center report on 18 April 2023 indicates that the median age of MPOX-infected individuals in Spain was 37 years (IQR 31–44). Additionally, the report states that 98.7% of those infected were male. Excluding cases with missing information, 95.5% of patients were identified as MSM [23]. Multicenter case series studies demonstrate consistent findings. For instance, a study carried out in 16 non-endemic countries for MPOX, which was published in late August 2022, reported that the median age of individuals infected was 38 years, with more than 99% of cases occurring in males, and 98% of infected individuals being MSM [10]. Another study, utilizing data from the GeoSentinel surveillance system and including patients from 15 countries, revealed that 99% of cases with available data were MSM, with a median age of 37 years (IQR 32–43) [24]. Similar patterns are evident within specific countries. For example, in a UK study, the median age was 37.8 years, and 95% were MSM [25]. Another study conducted in a university hospital in Marseille (southern France) studied MPOX patients, who had a median age of 36 years (IQR 30–42), and 92% were MSM [26]. Another study in a hospital in Paris showed a median age of 35 years (IQR 30–41); 99% were male, and 95% were MSM [27].

Worldwide, according to WHO data, as of 18 April 2023, the median age of MPOX patients is 34 years (IRQ 29–41 years), with 96.4% being male and 84.1% of cases with available data being MSM. According to the same source, cases of MPOX in endemic regions of Africa follow a different demographic distribution. As of 21 March 2023, 1448 confirmed cases of MPOX had been reported in the region, resulting in 18 deaths. These cases account for 2% of global cases, yet 16% of global deaths. Of the reported cases, 401 have been detailed, revealing that 61.1% were males and 38.9% were females, with a median age of 23 (IQR 8–35). Of these cases, 40.6% were under 18 years of age. Although sexual transmission has traditionally been less common in the endemic area compared to non-endemic countries, there are no available details on the transmission route for this outbreak in these regions [19].

In our hospital, none of the patients studied had traveled to Africa within the 21 days before symptom onset. However, all ten patients who identified close contact with an MPOX-positive or probable case were themselves MPOX positive. Therefore, most patients who satisfied epidemiological criteria had been in a high-risk sexual context in the 21 days before the onset of their symptoms. In a study that provides epidemiological features of MPOX cases reported during May and June 2022 in Spain, of 440 studied individuals who had available information, 101 were reported to be close contacts of confirmed or probable case patients. Furthermore, 332 (85.8% of MPOX positives who had available information) reported, as the mechanism of transmission, intimate and prolonged contact during sex [28].

In our analysis, we found that more than half of those who had no partner or had an open partner, despite being at risk of acquiring a sexually transmitted disease, never used a condom during oral sex, regardless of whether they were MPOX positive or negative and whether or not they had an HIV infection, based on data from the 12 months before the interview. However, only 11% of study participants never used condoms during anal sex. This difference in condom use depending on sexual practice is a possible topic for further research, as it dramatically impacts the prevention of transmission of sexually transmitted diseases.

Also notable was the use of mobile apps to arrange sexual encounters. In our study, half of the patients reported using these apps within the 12 months before the interview, regardless of whether they were MPOX positive or negative and whether or not they had HIV. In a study about 185 MPOX-positive patients in Spain, 55% had used social networks to meet partners [27]. A rapid risk assessment by the European Center for Disease Prevention and Control (ECDC) on 23 May 2022 proposes using these applications to transmit prevention messages to its users [17].

### 4.2. Clinical Discussion

In the present study, the proportion of MPOX infection among patients with clinical suspicion of the disease admitted to our hospital during the study period was 72.9%. Therefore, the suggested ECDC diagnostic criteria for “suspected” case definition was valid and valuable in our hospital, as nearly 100% of confirmed cases fulfilled them. However, up to 20% of negatives did not meet them and were still tested for MPOX. We believe that this attitude was correct and justified by the epidemiological context.

We describe the clinical characteristics of confirmed MPOX cases compared to negative patients. Positive cases were found to have a higher frequency of some general features, such as fever, dysuria, and rectal pain, and these appeared at the same time as the eruption or between one and two days earlier. Recent studies of this latest MPOX outbreak in Spain reveal systemic symptoms in most patients with the infection, indicating an invasive phase of illness [29]. However, in our study, negative cases had similar rates of headache, asthenia, and arthro-myalgias, so these systemic symptoms do not help us to distinguish a patient with MPOX infection clearly. In fact, in a London cohort, almost half (47.2%) of the patients had exclusively mucocutaneous manifestations at presentation or developed systemic symptoms after, rather than preceding, the onset of lesions [9]. In another London cohort, fatigue, asthenia, or lethargy were found in only 67% of individuals, and fever or febrile chills were reported in 57% [30]. This lack of specificity of systemic symptoms can be challenging when evaluating potential patients with MPOX [31].

On the contrary, inguinal adenopathy was a suggestive factor of MPOX infection as it was significantly found in cases more than in negatives. Furthermore, in the current outbreak, regional lymphadenopathies have been described in the lymph catchment area of lesions, in contrast with generalized swelling of the lymph nodes observed in previous reports of MPOX virus infections in endemic countries [32]. In fact, according to recent studies, more than 90% of MPOX infections can develop lymphadenopathy in the early stage of the disease, which is also considered a meaningful sign to distinguish MPOX from other infectious skin diseases [33].

A very recent systematic review found that fever, lymphadenopathy (mainly inguinal lymphadenopathy), and typical skin lesions (mainly anogenital areas) were the main clinical features of this current MPOX outbreak. In our study, fever, myalgias, and lymphadenopathy were the most common prodromal symptoms in MPOX cases [31].

Pustules, pseudo-pustules, and ulcerating lesions were more prevalent in positive patients, whereas negative patients had a greater prevalence of erythematous papules and vesicles. This fact supports the idea of suspecting MPOX infection when pustules, especially pseudo-pustules, are present, mainly in genital areas, within a favorable epidemiological context [34]. In the Spanish cohort, pseudo-pustules constituted the most prevalent primary lesions in the likely inoculation areas, spreading pustules in distant regions. The macular rash was uncommon in our study, in both positive and negative patients, in line with the current literature [29]. When macular lesions appear, STIs and drug eruptions must be included in the differential diagnosis. Recent reports suggest that skin lesions can skip morphologic phases, progressing from papule to ulcer and including multiple lesion types at any point during the illness [35].

The number of lesions appears to be fewer in this current outbreak compared to previous ones from endemic countries [27], and we found similar results in our institution, as having more than 25 lesions was highly infrequent and, in contrast, some cases even presented with a solitary lesion, mainly in genital areas.

Perianal, buttocks, hands, feet, and genital (penis and perianal) areas were more involved in cases than non-infected patients. It has been suggested that lesions in this current outbreak started at the point of contact from which the virus has been transmitted [34]. As the main route of infection in this current outbreak has been the sexual pathway, our impression is, by recent studies, that skin lesions initiated and first concentrated at the site of sexual contact with virus inoculation in the area and subsequent dissemination of lesions during the invasive phase [36]. Furthermore, it has been postulated that mild trauma in the pubic, inguinal, and perianal regions during sexual intercourse might cause local vasodilation and a higher density of skin lesions in that particular region (also known as the garter effect) [32]. In a recent systematic review, it was found that limbs were the most frequently affected areas (77% of patients), followed by anogenital lesions (66%) [34]. Classical MPOX eruption, described in previous outbreaks, usually started in the face and neck in 62–97% of cases, with ulterior spreading to the trunk, limbs, hands, and feet in 81–100% of individuals. In contrast, only two thirds of patients had genital involvement [37].

The main complications in our MPOX cases were similar to those described in other articles [30]. Genital lesions were sometimes complicated with coalescing ulceration, penile swelling with edema, and proctitis with severe rectal pain. In addition, we had several cases of secondary bacterial infection with cellulitis and local pain. A small number of ulcerated lesions led to residual atrophic or fibrotic scarring lesions. The scarring has been found in 13% of the registry cases in a recent study representing a potential long-term sequela.

Additionally, aphthous ulcers in the oropharynx produced tonsillitis with significant pain and even dysphagia, requiring assessment by an otorhinolaryngologist to discard a potential compromise of the airway. In these complicated cases, analgesia and early antibiotic therapy were administered with subsequent improvement in all patients [38]. We did not register any serious complications or deaths related to the infection. However, bronchopneumonia, sepsis, encephalitis, myocarditis, keratitis with visual loss, and rectal wall perforations have been described in previous reports [39]. Our hospitalization rate is close to the 8–13% seen in previous studies [31].

Our data support previously reported results pointing out that concurrent STIs are common in persons with MPOX [40]. It has been suggested that STIs could become a facilitator factor for MPOX infection, and it is essential to conduct an STI screening in all cases [29].

Regarding negative subjects, syphilis and varicella infection were the most common causes of MPOX mimics. Varicella is one of the most likely differential diagnoses. However, it mainly produces smaller liquid-filled lesions (vesicles) instead of pustules (pus-filled lesions) or pseudo-pustules, and various stages of lesions can be seen in the same patient. Moreover, lesions often concentrate on the head and trunk, highly infrequently involving palms, soles, and anogenital areas, which are typically affected in MPOX cases.

As previously mentioned, the painful lymphadenopathy in the prodromal stage is crucial to differentiating MPOX infection from other infectious diseases such as varicella, smallpox, or chickenpox.

Finally, secondary syphilis, which develops rapidly after the initial chancre, could be easily confused with MPOX [41].

### 4.3. HIV-Coinfection and Smallpox Vaccination

The irruption of MPOX in a Western urban context outside its rural environment in Africa has been especially evident in a high percentage of people living with HIV (PLWHIV). In Europe, according to TESSy data updated to March 2023, 37.8% of patients with MPOX had HIV [42]. In other systematic reviews and meta-analyses, this percentage varies between 30.18% to 42.2% [31,39,43]. This percentage is almost twice as high in our hospital in Madrid, Spain, at 71.9%. A possible explanation may be easy access to the healthcare system and the infectious disease physicians of PLWHIV under clinical follow-up in our hospital. This could justify closer monitoring than patients without HIV.

Chronic HIV infection could predispose to more frequent and severe viral infections than the general population. This is mainly due to reduced CD4 cell counts, significantly below 200 cells/mm^3^. In this sense, late diagnosis with a CD4 count below 350 cells/mm^3^ remains an unresolved problem in our population, occurring in more than half of the cases (59.3%). Moreover, 9.1% met AIDS criteria at HIV diagnosis. Unfortunately, these alarming data on late infection are far from unusual, being similar to those observed in the rest of Spain and Europe [44]. Despite these data, in MPOX diagnosis, the immunovirological status is optimal in most cases. This is explained by high adherence to antiretroviral therapy (ART). Thus, only four patients (6.9%) had a detectable viral load at MPOX diagnosis. In addition, the median CD4 cell count at that time was 607.0 cells/mm^3^.

During an outbreak caused by a virus where a significant percentage of patients have previous HIV infection, one might ask whether the course of MPOX differs between these patients and those who do not have HIV. Looking at prior outbreaks, especially in countries where MPOX is an endemic infection, it is clear that the course of the disease is more severe in patients with a history of HIV infection [10,45]. Furthermore, this severity has been related to a higher number and more widespread skin lesions, a more prolonged and more protracted course of the disease, a higher rate of superinfection of skin lesions, a higher rate of severe complications such as encephalitis, and higher mortality [37,46,47].

However, the recent outbreak has affected many countries with very different socio-sanitary characteristics from those where MPOX is endemic. Numerous descriptive studies, reviews, and meta-analyses have found no significant association between having HIV and having more severe MPOX [10,29,30,45]. The most plausible explanation is that most of these patients have easy access to ART (according to Qi Liu’s meta-analysis, a median of 99%), which means that the immunovirological status of these patients is optimal. They are virtually not immunosuppressed [31,48]. Thus, the mean CD4 cell counts at the time of diagnosis of MPOX in the different studies are similar to those found in our population: 680 to 713 cells/mm^3^ [10,24,29] or 677 cells/mm^3^ [31]. Moreover, the other aspect is the presence, at the moment of MPOX diagnosis, of an undetectable HIV viral load (<50 copies/mL) in about 95% of patients, as found in our population [10,24,29,31]. This access to ART and optimal immunovirological status is not fulfilled in the same way in countries where MPOX is endemic. All of this justifies why we have not found significant differences in the course of the disease in the current outbreak as in previous episodes.

However, it is noteworthy that some studies have found a difference in the course of disease in HIV-positive patients. For example, in a Lancet observational study looking at 226 cases from 15 different countries, having HIV was associated with a higher likelihood of developing diarrhea, perianal lesions, and a higher total number of lesions. In addition, those with CD4 < 500 cells/mm^3^ had even more skin lesions than those with >500 cells/mm^3^ [24]. Another US study involving 1969 MPOX cases showed that those with HIV were more likely to have proctalgia, rectorrhagia, rectal tenesmus, pus-emitting stools, and proctitis [49]. Our study found no differences between PLWHIV and HIV-negative groups in the number, size, or morphology of skin lesions. However, we found a tendency for greater perianal involvement in PLWHIV (21.7%) compared to those without HIV (3.7%). This was especially manifest in those with poor immunological status related to inadequate control of HIV infection. In this subgroup, we observed perianal involvement in 30.3% vs. 0% in those with CD4 counts over 500 cells/mm^3^. In addition, among patients with well-controlled HIV, facial involvement is 0% compared to 24.2% in those with poor immunological status. Regarding the course of the disease, we also found some notable differences. Eight of the nine patients with bacterial superinfection of skin lesions had a history of HIV infection. Moreover, five of the six patients who required hospitalization had a history of HIV infection. As a final difference in the PLWHIV group, higher C-reactive protein levels were observed analytically compared to HIV-negative patients, with a median of 46.3 mg/L vs. 14.1 mg/L (*p* = 0.026).

Finally, it is exciting to discuss the role of smallpox vaccination in the recent outbreak of MPOX. First, it is essential to remember that the monkeypox virus belongs to the family Poxviridae and the genus Orthopoxvirus [50]. Orthopoxvirus includes the famous and now-eradicated smallpox, cowpox, camelpox, and vaccinia virus [46]. There is a cross-reaction at the immunological level due to two factors. Firstly, the immune response, both humoral and cellular, is directed against many structural proteins. The second factor is the high similarity in immunologically highly relevant proteins between Orthopoxvirus species. It is, therefore, no coincidence that an increase in outbreaks of monkeypox virus infection in recent decades has coincided with a reduction in the prevalence of smallpox immunity in the world population [51]. Nevertheless, immunological cross-reactivity also explains another exciting observation. In the MPOX outbreaks between 1980–1984 and 2006–2007 in the Central Republic of Congo, reductions in the risk of acquiring the disease of 85% and 80.7%, respectively, were observed in those individuals who were vaccinated against smallpox [51]. In addition, numerous reports suggest that the course of the disease is modified by vaccination, with a less extensive skin rash, fewer lesions, less systemic symptomatology, and less risk of developing complications [45,51]. Therefore, considering whether something similar occurred during the recent outbreak is of great interest. However, the reality is that, to date, there are minimal data in the literature on the clinical effectiveness of the vaccine in modifying the course of MPOX during the 2022 outbreak [52]. Our study found a smallpox vaccination rate among confirmed MPOX cases of 15.0%, slightly higher than that found in other studies (9–12%) [10,24,29,31]. Significantly, vaccinated patients had less fever during the disease than unvaccinated patients (14.3% vs. 51.6%, *p* = 0.025). Unfortunately, we could not establish an analytical correlation for this clinical finding due to a lack of data. In addition, we observed a tendency to have fewer pustule-like lesions among previously vaccinated patients compared to non-vaccinated patients (21.4% vs. 51.6%, *p* = 0.08). Finally, none of our patients who experienced bacterial superinfection of skin lesions or had to be admitted were vaccinated. Given the vaccine’s efficacy in preventing infection and the development of a more severe disease course, this strategy is highly recommended.

## 5. Conclusions

In conclusion, the MPOX 2022 outbreak in our area has been related to sexual intercourse among MSM, with no severe clinical cases nor apparent differences in HIV and non-HIV patients.

## Figures and Tables

**Table 1:** Epidemiological characteristics.

		MPOX			MPOX Confirmed Cases		
	Overall	Yes	No	*p*-Value	PLWHIV	HIV-Negative	*p*-Value
**N**	133	100	33		71	28	
**Age (years) (median [IQR])**	33.0 [29.0, 41.0]	33.0 [30.0, 42.0]	33.0 [27.0, 36.0]	0.079	33.5 [30.0, 42.0]	33.0 [29.0, 40.0]	0.711
**Sex (%) Male**	124 (96.1)	97 (99.0)	27 (87.1)	0.125	70 (100.0)	26 (96.3)	0.325
**Relationship status (%)**							
No partner	58 (55.2)	48 (54.5)	10 (58.8)	0.927	35 (55.6)	12 (50.0)	0.636
Closed couple	32 (30.5)	27 (30.7)	5 (29.4)		20 (31.7)	7 (29.2)	
Open couple	15 (14.3)	13 (14.8)	2 (11.8)		8 (12.7)	5 (20.8)	
**Travel within 21 days before symptom onset (%)**	25 (24.3)	20 (23.3)	5 (29.4)	0.878	15 (24.6)	5 (20.8)	0.933
**Close contact with a probable or confirmed MPOX case (%)**	10 (9.8)	10 (11.8)	0 (0.0)	0.332	8 (13.3)	2 (8.3)	0.790
**Cohabitation with pets (%)**	33 (33.3)	27 (32.9)	6 (35.3)	1.000	20 (34.5)	7 (30.4)	0.931
**Sharing towels (%)**	35 (36.1)	29 (35.4)	6 (40.0)	0.890	17 (29.8)	12 (50.0)	0.140
**Sexual behavior in the last 12 months (%)**							
Men	87 (86.1)	75 (89.3)	12 (70.6)	0.026	56 (94.9)	18 (75.0)	0.015
Women	5 (5.0)	2 (2.4)	3 (17.6)		0 (0.0)	2 (8.3)	
Both	9 (8.9)	7 (8.3)	2 (11.8)		3 (5.1)	4 (16.7)	
**Number of sexual partners in the last 12 months (%)**							
1	16 (16.0)	13 (15.5)	3 (17.6)	0.782	10 (16.9)	3 (12.5)	0.098
2–5	36 (36.0)	30 (35.7)	6 (35.4)		17 (28.9)	12 (50.0)	
6–9	10 (10.0)	11 (10.8)	1 (5.9)		12 (11.9)	2 (8.3)	
10–20	26 (26.0)	21 (25.0)	5 (29.4)		14 (23.7)	7 (29.2)	
>20	13 (13.0)	11 (13.1)	2 (11.8)		11 (18.7)	0 (0.0)	
**Saunas (%)**	16 (16.0)	14 (16.7)	2 (11.8)	0.842	14 (23.7)	0 (0.0)	0.022
**Parties (%)**	15 (15.0)	12 (14.1)	3 (17.6)	1.000	11 (18.3)	1 (4.2)	0.183
**Use of an app or social networks for sexual encounters (%)**	53 (52.5)	45 (53.6)	8 (47.1)	0.781	31 (52.5)	14 (58.3)	0.813
**Sex with sex workers (%)**	5 (5.0)	5 (6.0)	0 (0.0)	0.649	3 (5.2)	2 (8.3)	0.970
**Unprotected sex in the last 12 months**							
**Oral sex (%)**							
Never	10 (10.1)	9 (10.8)	1 (6.2)	0.728	6 (10.3)	3 (12.5)	0.905
Sometimes	37 (37.4)	30 (36.1)	7 (43.8)		20 (34.5)	9 (37.5)	
Always	52 (52.5)	44 (53.0)	8 (50.0)		32 (55.2)	12 (50.0)	
**Vaginal sex (%)**							
Never	6 (50.0)	3 (42.9)	3 (60.0)	1.000	2 (66.7)	1 (25.0)	0.459
Sometimes	4 (33.3)	3 (42.9)	1 (20.0)		1 (33.3)	2 (50.0)	
Always	2 (16.7)	1 (14.3)	1 (20.0)		0 (0.0)	1 (25.0)	
**Anal sex (%)**							
Never	36 (36.7)	32 (39.0)	4 (25.0)	0.519	19 (32.8)	13 (56.5)	0.113
Sometimes	51 (52.0)	41 (50.0)	10 (62.5)		31 (53.4)	9 (39.1)	
Always	11 (11.2)	9 (11.0)	2 (12.5)		8 (13.8)	1 (4.3)	
**Practice of chemsex (%)**	23 (23.0)	19 (22.9)	4 (23.5)	1.000	16 (27.6)	3 (12.5)	0.236
**Sexual behavior in last 21 days (%)**							
Men	84 (90.3)	70 (94.6)	11 (68.8)	0.007	53 (98.1)	19 (86.4)	0.062
Women	5 (5.4)	2 (2.7)	3 (18.8)		0 (0.0)	2 (9.1)	
Both	4 (4.3)	2 (2.7)	2 (12.5)		1 (1.9)	1 (4.5)	
**Saunas in the last 21 days (%)**	7 (7.1)	6 (7.3)	1 (5.9)	1.000	6 (10.5)	0 (0.0)	0.235
**Parties in the last 21 days (%)**	12 (12.0)	10 (12.0)	2 (11.8)	1.000	9 (15.5)	1 (4.2)	0.290
**Use of an app or social network for sexual encounters in the last 21 days (%)**	32 (32.3)	28 (34.1)	4 (23.5)	0.572	23 (40.4)	5 (20.8)	0.153
**Sex with sex workers (%)**	3 (3.1)	2 (2.5)	1 (5.9)	1.000	1 (1.8)	1 (4.2)	1.000
**Use of sex toys (%)**	8 (8.4)	6 (7.6)	2 (12.5)	0.915	6 (11.1)	0 (0.0)	0.215
**Unprotected sex in the last 21 days**							
**Oral sex (%)**							
Never	14 (16.1)	12 (16.4)	2 (14.3)	0.950	7 (13.7)	4 (19.0)	0.850
Sometimes	21 (24.1)	18 (24.7)	3 (21.4)		13 (25.5)	5 (23.8)	
Always	52 (59.8)	43 (58.9)	9 (64.3)		31 (60.8)	12 (57.1)	
**Vaginal sex (%)**							
Never	7 (63.6)	4 (66.7)	3 (60.0)	0.565	2 (100.0)	2 (50.0)	0.472
Sometimes	2 (18.2)	1 (16.7)	1 (20.0)		0 (0.0)	1 (25.0)	
Always	2 (18.2)	1 (16.7)	1 (20.0)		0 (0.0)	1 (25.0)	
**Anal sex (%)**							
Never	32 (36.4)	26 (35.6)	6 (40.0)	0.970	16 (31.4)	10 (47.6)	0.410
Sometimes	38 (43.2)	32 (43.8)	6 (40.0)		24 (47.1)	7 (33.3)	
Always	18 (20.5)	15 (20.5)	3 (20.0)		11 (21.6)	4 (19.0)	
**Practice of “chemsex” in the last 21 days (%)**	15 (15.3)	13 (16.0)	2 (11.8)	0.892	11 (19.6)	2 (8.3)	0.355

Medians were compared using the Mann–Whitney U test. Frequencies were compared using the Chi-Square test and Fisher’s exact test.

**Table 2:** Skin lesions characteristics.

		MPOX			MPOX Confirmed Cases		
	Overall	Yes	No	*p*-Value	PLWHIV	HIV-Negative	*p*-Value
	133	100	33		71	28	
**Type of skin lesion (%)**							
Macule	8 (6.0)	6 (6.0)	2 (6.1)	1.000	5 (7.0)	1 (3.6)	0.854
Papule	55 (41.4)	40 (40.0)	15 (45.5)	0.728	29 (40.8)	10 (35.7)	0.809
Vesicle	52 (39.1)	38 (38.0)	14 (42.4)	0.806	27 (38.0)	10 (35.7)	1.000
Pustule	54 (40.6)	**45 (45.0)**	9 (27.3)	0.111	35 (49.3)	9 (32.1)	0.186
Erosion/ulceration	42 (31.6)	36 (36.0)	6 (18.2)	0.090	25 (35.2)	11 (39.3)	0.883
Crust	28 (21.1)	22 (22.0)	6 (18.2)	0.826	12 (16.9)	9 (32.1)	0.162
**Number of skin lesions (%)**							
Single lesion	4 (3.0)	4 (4.0)	0 (0.0)	0.563	1 (1.4)	3 (10.7)	0.121
2 to 25 skin lesions	73 (54.9)	**54 (54.0)**	19 (57.6)	0.876	40 (56.3)	14 (50.0)	0.729
25 to 100 lesions	5 (3.8)	1 (1.0)	**4 (12.1)**	**0.017**	1 (1.4)	0 (0.0)	1.000
Unknown	51 (38.3)	41 (41.0)	10 (30.3)	0.374	29 (40.8)	11 (39.3)	1.000
**Size of skin lesions (%)**							
<1 mm	3 (2.3)	2 (2.0)	1 (3.0)	1.000	2 (2.8)	0 (0.0)	0.917
1 to 5 mm	33 (24.8)	24 (24.0)	9 (27.3)	0.885	19 (26.8)	5 (17.9)	0.502
>5 mm	17 (12.8)	13 (13.0)	4 (12.1)	1.000	10 (14.1)	3 (10.7)	0.907
Unknown	87 (65.4)	68 (68.0)	19 (57.6)	0.379	46 (64.8)	21 (75.0)	0.459
**Evolution of skin lesions (%)**							
Favorable	36 (27.1)	29 (29.0)	7 (21.2)	0.518	21 (29.6)	7 (25.0)	0.835
Bacterial superinfection	9 (6.8)	9 (9.0)	0 (0.0)	0.166	**8 (11.3)**	**1 (3.6)**	0.417
Residual lesion	7 (5.3)	6 (6.0)	1 (3.0)	0.831	4 (5.6)	2 (7.1)	1.000
Unknown	83 (62.4)	58 (58.0)	25 (75.8)	0.105	41 (57.7)	17 (60.7)	0.965
**Hospitalized (stay of at least one night) (%)**	**6 (4.5)**	6 (4.5)	6 (6.2)	0 (0.0)	**5 (7.4)**	**1 (3.7)**	0.321
**Death (%)**	**0 (0.0)**	0 (0.0)	0 (0.0)	0 (0.0)	-	0 (0.0)	0 (0.0)

Frequencies were compared using the Chi-Square test and Fisher’s exact test. We highlighted in bold some of the most significant features.

**Table 3:** HIV-related data of PLWHIV.

	Overall	Yes	No	*p*-Value
	133	100	33	
**Previous HIV diagnosis (%)**	82 (62.1)	71 (71.0)	11 (33.3)	<0.001
**CD4 at diagnosis (median [IQR])**	284.0 [192.0, 462.0]	252.5 [170.5, 416.8]	481.0 [303.5, 574.0]	0.040
**HIV CV at diagnosis (median [IQR])**	182,700.0 [23,395.8, 410,900.0]	165,000.0 [15,326.5, 398,800.0]	200,400.0 [116,850.0, 1,250,200.0]	0.547
**Late diagnosis (< 350 CD4 at diagnosis or AIDS-defining event at diagnosis) (%)**	**32 (59.3)**	30 (63.8)	2 (28.6)	0.166
**AIDS at diagnosis (%)**	**6 (9.1)**	6 (10.5)	0 (0.0)	0.672
**Time to ART initiation (weeks) (median [IQR])**	12.0 [4.0, 216.0]	20.0 [4.5, 249.0]	4.0 [2.0, 10.0]	0.184
**Time to achieve undetectable CV (weeks) (median [IQR])**	20.0 [12.0, 48.0]	19.0 [12.0, 49.0]	24.0 [8.0, 28.0]	0.591
**CD4 around Monkeypox (median [IQR])**	**617.0 [436.0, 910.0]**	607.0 [394.0, 914.0]	754.5 [509.2, 869.5]	0.597
**Detectable VL around Monkeypox (%)**	**4 (6.1)**	4 (6.9)	0 (0.0)	1.000
**VL around Monkeypox (median [IQR])**	76.5 [65.0, 89.8]	78.2 (29.7)	-	-
**Coinfections**				
HBV serology: HBs Ag (%)	18 (13.5)	16 (16.0)	2 (6.1)	0.278
HBc IgM (%)	11 (8.3)	11 (11.0)	0 (0.0)	0.123
HBc IgG (%)	33 (24.8)	31 (31.0)	2 (6.1)	0.011
HBs Ac (%)	62 (46.6)	53 (53.0)	9 (27.3)	0.021
Active HCV (%)	5 (6.7)	4 (6.1)	1 (11.1)	1.000

AIDS = acquired immunodeficiency syndrome; ART = antiretroviral therapy; CV = viral load; HBV = hepatitis B virus; HCV = hepatitis C virus. Medians were compared using the Mann–Whitney U test. Frequencies were compared using the Chi-Square test and Fisher’s exact test. We highlighted in bold some of the most significant features.

**Table 4:** Clinical characteristics of the MPOX depend on immunovirological HIV control and vaccination status against smallpox.

	Good Immunovirological HIV Control			Vaccinated against Smallpox		
	Yes	No	*p*-Value	Yes	No	*p*-Value
	7	33		15	66	
**Days between the onset of symptoms and skin lesions (mean (SD))**	7.7 (12.0)	2.2 (4.1)	0.315	2.2 (7.0)	1.7 (3.5)	0.542
**Clinical manifestations (%)**						
Headache	1 (14.3)	11 (33.3)	0.586	3 (21.4)	19 (29.7)	0.769
Fever	5 (71.4)	15 (45.5)	0.405	2 (14.3)	33 (51.6)	0.025
Myalgias, dysthermic sensation	2 (28.6)	16 (48.5)	0.587	6 (42.9)	29 (45.3)	1.000
Arthralgias	0 (0.0)	6 (18.2)	0.522	1 (7.1)	6 (9.4)	1.000
Odynophagia	1 (14.3)	6 (18.2)	1.000	2 (14.3)	5 (7.8)	0.801
Asthenia	0 (0.0)	11 (33.3)	0.184	4 (28.6)	17 (26.6)	1.000
Inguinal adenopathies	4 (57.1)	15 (45.5)	0.884	5 (35.7)	34 (53.1)	0.376
Axillary adenopathies	1 (14.3)	1 (3.0)	0.775	0 (0.0)	2 (3.1)	1.000
Urethral exudate, dysuria	0 (0.0)	1 (3.0)	1.000	0 (0.0)	3 (4.7)	0.953
Rectal discharge, anal pain	2 (28.6)	2 (6.1)	0.267	3 (21.4)	7 (10.9)	0.534
Pruritus	2 (28.6)	3 (9.1)	0.432	3 (21.4)	7 (10.9)	0.534
**Type of skin lesion (%)**						
Macule	0 (0.0)	3 (9.1)	0.968	1 (7.1)	5 (7.8)	1.000
Papule	1 (14.3)	15 (45.5)	0.269	4 (28.6)	27 (42.2)	0.521
Vesicle	4 (57.1)	17 (51.5)	1.000	6 (42.9)	23 (35.9)	0.857
Pustule	3 (42.9)	17 (51.5)	1.000	3 (21.4)	33 (51.6)	0.080
Erosion, ulceration	2 (28.6)	12 (36.4)	1.000	5 (35.7)	22 (34.4)	1.000
Crust	2 (28.6)	8 (24.2)	1.000	4 (28.6)	14 (21.9)	0.850
**Number of skin lesions (%)**						
Single lesion	1 (14.3)	0 (0.0)	0.386	1 (7.1)	3 (4.7)	1.000
2 to 25 skin lesions	6 (85.7)	20 (60.6)	0.407	9 (64.3)	30 (46.9)	0.376
25 to 100 lesions	0 (0.0)	1 (3.0)	1.000	0 (0.0)	1 (1.6)	1.000
Unknown	0 (0.0)	12 (36.4)	0.146	4 (28.6)	30 (46.9)	0.340
**Size of skin lesions**						
<1 mm	1 (14.3)	1 (3.0)	0.775	0 (0.0)	1 (1.6)	1.000
1 to 5 mm	4 (57.1)	10 (30.3)	0.360	3 (21.4)	15 (23.4)	1.000
>5 mm	2 (28.6)	6 (18.2)	0.917	2 (14.3)	10 (15.6)	1.000
Unknown	1 (14.3)	19 (57.6)	0.096	10 (71.4)	43 (67.2)	1.000
**Location of skin lesions (%)**						
Facial	0 (0.0)	8 (24.2)	0.349	3 (21.4)	19 (29.7)	0.769
Scalp	0 (0.0)	1 (3.0)	1.000	0 (0.0)	8 (12.5)	0.363
Neck	2 (28.6)	1 (3.0)	0.123	0 (0.0)	4 (6.2)	0.771
Oral cavity	0 (0.0)	3 (9.1)	0.968	0 (0.0)	4 (6.2)	0.771
Genital	3 (42.9)	18 (54.5)	0.884	7 (50.0)	35 (54.7)	0.982
Groin or pubis	2 (28.6)	12 (36.4)	1.000	2 (14.3)	19 (29.7)	0.399
Perianal	0 (0.0)	10 (30.3)	0.23	3 (21.4)	11 (17.2)	1.000
Chest	1 (14.3)	7 (21.2)	1.000	3 (21.4)	21 (32.8)	0.606
Abdomen	0 (0.0)	7 (21.2)	0.427	1 (7.1)	22 (34.4)	0.089
Back	0 (0.0)	7 (21.2)	0.427	3 (21.4)	18 (28.1)	0.858
Arm	1 (14.3)	14 (42.4)	0.334	6 (42.9)	23 (35.9)	0.857
Hands	1 (14.3)	6 (18.2)	1.000	1 (7.1)	10 (15.6)	0.688
Legs	1 (14.3)	10 (30.3)	0.692	1 (7.1)	23 (35.9)	0.073
Feet	0 (0.0)	1 (3.0)	1.000	1 (7.1)	1 (1.6)	0.792
Soles of feet	0 (0.0)	1 (3.0)	1.000	1 (7.1)	4 (6.2)	1.000
Palm of hands	0 (0.0)	1 (3.0)	1.000	1 (7.1)	1 (1.6)	0.792
Ocular	0 (0.0)	1 (3.0)	1.000	0 (0.0)	1 (1.6)	1.000
Buttocks	2 (28.6)	3 (9.1)	0.432	2 (14.3)	6 (9.4)	0.95
**Evolution of skin lesions (%)**						
Favorable	2 (28.6)	10 (30.3)	1.000	4 (28.6)	21 (32.8)	1.000
Superinfection	1 (14.3)	4 (12.1)	1.000	**0 (0.0)**	**8 (12.5)**	0.363
Residual lesion	1 (14.3)	2 (6.1)	1.000	1 (7.1)	4 (6.2)	1.000
Unknown	5 (71.4)	18 (54.5)	0.689	9 (64.3)	33 (51.6)	0.569
**Hospitalized (stay of at least one night) (%)**	1 (14.3)	2 (6.1)	1.000	**0 (0.0)**	**5 (7.8)**	0.632
**Death (%)**	0 (0.0)	0 (0.0)	-	0 (0.0)	0 (0.0)	-

Frequencies were compared using the Chi-Square test and Fisher’s exact test. We highlighted in bold some of the most significant features.

## Data Availability

The data are available upon request from the corresponding author.

## Abbreviations

The following abbreviations are used in this manuscript:

HIV	Human immunodeficiency virus
PLWHIV	patients living with human immunodeficiency virus
MPOX	monkeypox
“Chemsex”	chemical sex
MSM	men who have sex with men
WHO	World Health Organization
PHEIC	Public Health Emergency of International Concern
PCR	polymerase chain reaction
STI	sexually transmitted infections
IQR	interquartile range
ECDC	European Center for Disease Prevention and Control
TESSy	The European Surveillance System
ART	antiretroviral therapy

## Appendix A

**Table A1:** Analytical data.

		MPOX			MPOX-Confirmed Cases		
	Overall	Yes	No	*p*-Value	PLWHIV	HIV-Negative Cases	*p*-Value
	133	100	33		71	28	
**Biochemistry (median [IQR])**							
**Creatinine**	0.9 [0.9, 1.0]	0.9 [0.9, 1.0]	0.9 [0.8, 1.0]	0.560	0.9 [0.9, 1.1]	0.9 [0.8, 0.9]	0.390
**Sodium**	139.0 [138.0, 140.0]	139.0 [138.0, 141.0]	139.0 [139.0, 139.0]	0.676	139.0 [137.5, 141.5]	139.0 [138.2, 139.0]	0.831
**Potassium**	4.3 [4.1, 4.5]	4.3 [4.1, 4.5]	4.5 [4.3, 4.6]	0.267	4.2 [4.0, 4.4]	4.4 [4.2, 4.7]	0.131
**Calcium**	9.4 [9.3, 9.7]	9.4 [9.2, 9.8]	9.6 [9.5, 9.6]	0.849	9.4 [9.2, 9.8]	9.7 [9.6, 9.8]	0.342
**Magnesium**	1.9 [1.9, 2.0]	2.0 [1.9, 2.0]	1.9 [1.9, 1.9]	0.48	1.9 [1.9, 1.9]	2.0 [2.0, 2.0]	0.317
**Phosphorus**	3.1 [3.0, 3.4]	3.1 [2.9, 3.3]	3.2 [3.1, 3.4]	0.746	3.1 [2.8, 3.3]	3.4 [3.2, 3.6]	0.600
**C-reactive protein**	28.9 [19.8, 67.3]	32.4 [20.4, 80.1]	25.3 [18.1, 44.1]	0.600	**46.3** **[23.2, 85.0]**	**14.1** **[11.8, 23.3]**	**0.026**
**GOT**	29.0 [22.0, 43.5]	34.0 [22.0, 43.8]	27.0 [20.0, 27.0]	0.509	39.0 [26.0, 47.0]	22.0 [20.0, 22.0]	0.021
**GPT**	35.0 [25.0, 75.5]	35.5 [25.5, 76.2]	31.0 [25.0, 37.0]	0.604	43.0 [27.0, 79.0]	27.0 [22.0, 32.0]	0.106
**GGT**	34.0 [19.5, 54.5]	29.0 [19.8, 56.2]	39.0 [27.0, 48.0]	0.885	36.0 [21.0, 69.0]	20.0 [19.5, 27.0]	0.266
**Alkaline phosphatase**	89.5 [61.5, 104.8]	92.0 [62.0, 116.0]	87.0 [72.5, 90.5]	0.596	94.0 [74.0, 119.0]	55.5 [49.0, 68.0]	0.023
**LDH**	228.5 [196.5, 290.2]	228.5 [201.2, 285.0]	382.0 [271.5, 492.5]	0.900	239.0 [214.8, 290.2]	187.5 [182.8, 192.2]	0.092
**Bilirubin**	0.4 [0.3, 0.5]	0.4 [0.3, 0.5]	0.4 [0.4, 0.5]	0.727	0.4 [0.3, 0.5]	0.5 [0.4, 0.5]	0.552
**Total cholesterol**	145.0 [134.0, 156.0]	150.5 [135.5, 158.2]	139.0 [132.0, 140.0]	0.208	145.0 [130.0, 155.0]	159.0 [153.5, 162.5]	0.243
**LDL**	83.0 [74.0, 101.0]	88.5 [76.8, 101.8]	64.0 [56.5, 71.0]	0.078	83.0 [74.5, 95.5]	102.0 [97.5, 105.5]	0.102
**HDL**	36.0 [31.0, 45.0]	35.0 [30.2, 44.8]	43.0 [39.5, 47.5]	0.255	34.0 [30.0, 42.0]	44.0 [37.5, 44.5]	0.585
**Triglycerides**	93.0 [85.0, 136.0]	91.5 [82.0, 135.0]	133.0 [110.5, 161.0]	0.378	93.0 [87.5, 141.0]	59.0 [56.5, 95.5]	0.242
**Hemogram (median [IQR])**							
**Hemoglobin**	14.9 [13.9, 15.7]	14.9 [13.9, 15.7]	14.9 [14.0, 15.5]	0.795	14.6 [14.0, 15.4]	15.7 [14.6, 15.7]	0.321
**Platelets**	297.5 [201.8, 200,000.0]	292.0 [201.0, 197,000.0]	340.0 [293.0, 213,000.0]	0.650	327.0 [214.5, 200,000.0]	197.0 [174.0, 279.5]	0.078
**Leukocytes**	5720.0 [8.3, 7232.5]	5810.0 [8.1, 7850.0]	5540.0 [9.5, 6960.0]	0.880	6110.0 [8.0, 7667.5]	5360.0 [9.8, 7505.0]	0.826
**Neutrophils**	3050.0 [5.4, 4525.0]	3400.0 [5.5, 4600.0]	2800.0 [5.4, 2800.0]	0.503	3500.0 [5.5, 4525.0]	2400.0 [5.8, 4350.0]	0.947
**Lymphocytes**	1300.0 [2.4, 2100.0]	1200.0 [2.4, 2100.0]	1900.0 [2.8, 2100.0]	0.619	1300.0 [2.2, 1975.0]	800.0 [3.0, 2100.0]	0.808
**Coagulation**							
**INR (median [IQR])**	1.1 [1.1, 1.2]	1.1 [1.1, 1.1]	1.2 [1.2, 1.2]	0.142	1.1 [1.1, 1.1]	1.1 [1.1, 1.1]	0.820

We highlighted in bold some of the most significant features.
